# Development of a clinical algorithm for treating urethral strictures based on a large retrospective single-center cohort

**DOI:** 10.12688/f1000research.9427.2

**Published:** 2017-04-24

**Authors:** Yuri Tolkach, Thomas Herrmann, Axel Merseburger, Martin Burchardt, Mathias Wolters, Stefan Huusmann, Mario Kramer, Markus Kuczyk, Florian Imkamp

**Affiliations:** 1 Department of Urology and Urological Oncology, Hannover Medical School, Hannover, Germany; 2 Institute of Pathology, University Hospital Bonn, Bonn, Germany; 3 Clinic of Urology, University Greifswald, Greifswald, Germany

**Keywords:** stricture, urethra, endoscopic treatment, urethrotomy

## Abstract

**Aim**: To analyze clinical data from male patients treated with urethrotomy and to develop a clinical decision algorithm.

**Materials and methods**: Two large cohorts of male patients with urethral strictures were included in this retrospective study, historical (1985-1995, n=491) and modern cohorts (1996-2006, n=470). All patients were treated with repeated internal urethrotomies (up to 9 sessions). Clinical outcomes were analyzed and systemized as a clinical decision algorithm.

**Results**: The overall recurrence rates after the first urethrotomy were 32.4% and 23% in the historical and modern cohorts, respectively. In many patients, the second procedure was also effective with the third procedure also feasible in selected patients. The strictures with a length ≤ 2 cm should be treated according to the initial length. In patients with strictures ≤ 1 cm, the second session could be recommended in all patients, but not with penile strictures, strictures related to transurethral operations or for patients who were 31-50 years of age. The third session could be effective in selected cases of idiopathic bulbar strictures. For strictures with a length of 1-2 cm, a second operation is possible for the solitary low-grade bulbar strictures, given that the age is > 50 years and the etiology is not post-transurethral resection of the prostate. For penile strictures that are 1-2 cm, urethrotomy could be attempted in solitary but not in high-grade strictures.

**Conclusions**: We present data on the treatment of urethral strictures with urethrotomy from a single center. Based on the analysis, a clinical decision algorithm was suggested, which could be a reliable basis for everyday clinical practice.

## Introduction

Urethral stricture disease is a common problem in urological practice ^1, 2^. In general, the following three main types of treatment are applied in patients with urethral stricture disease: urethral dilatation, endoscopic treatment (urethrotomy) and urethroplasty ^3^, with urethrotomy being the most frequently applied and mastered by almost all urologists ^4, 5^. The reported success rates for endoscopic urethrotomy widely range from 32% to 73.1% with an understudied long-term success rate ^2, 6, 7^.

The relative easiness of the procedure and direct initial effect after procedure in all patients could explain the misuse of urethrotomy in patients, in whom recurrence after treatment is an obvious reality. The guidelines issued by the professional organizations do not generally recommend urethrotomy in patients with strictures longer than 1 cm or repeated urethrotomy sessions. Nevertheless, there is no strict evidence from prospective studies about the patient selection or repeated urethrotomy implementation or for the best treatment for stricture disease in general ^3^.

The aim of the current study was to analyze clinical data for a period of more than 20 years with endoscopic treatment of strictures in a large cohort of male patients as well as to develop a relevant and flexible clinical decision algorithm that could optimize the treatment of this patient group.

## Materials and methods

### Study design and data acquisition

The study was retrospective in nature. During the data acquisition period, clinical information was retrieved from medical records of male patients, who were initially treated in the urological clinic of Hannover Medical School with a diagnosis of urethral stricture using the urethrotomy in a period from 1985 to 2006.

### Patient cohorts

Two large cohorts of male patients with urethral strictures were included in this study, one historical cohort (Cohort I, treatment years 1985–1995, n=366) and one contemporary cohort (Cohort II, years 1996–2006, n=470) with a total of 961 patients. The patients were divided in these two cohorts with regard to the data quality (given the data acquisition was restrospective) with more consistent and full data in the “modern” Cohort II.

### Clinical characteristics

Clinical data, obtained from patient records, included the patient age at the moment of the first and following operations, stricture etiology, stricture localization, stricture length, stricture grade and number of strictures in every patient. The results of the preoperatively performed urethrography were obtained, whenever possible. The stricture length was calculated according to the urethrography images and partially derived from the urethroscopy protocols. A completely developed stricture grade classification was used. In all patients, the proportion of the minimal diameter in the stricture zone to the diameter of the normal urethra was calculated as a percent. Grade I was defined as lumen stenosis 33% or less, Grade II – 33–66% reduction in the lumen diameter, and Grade III – 66% and greater reduction. In some patients diameter of the urethra in the stricture zone was measured with the urethral catheter and further calculated as the percent of luminal stenosis. Traumatic stricture was defined as stricture, which has developed in clinically tight association with pelvic, perineal trauma / fracture of the pelvic bones. All information was entered in a database for subsequent statistical analysis.

### Treatment description

All patients were treated with respect to a urethral stricture using the internal cold-knife urethrotomy with the section at 12 o’clock while they were under general anesthesia. The patients with laser urethrotomy were not included in this study. Only patients without any prior treatment of urethral strictures were included in the study. Other urological conditions (e.g. benign prostatic hyperplasia, prostatitis, prostate cancer, medicaments) were not considered as exclusion criteria. A substantial number of the patients received multiple treatment sessions (up to 9 urethrotomies). The duration of the catheterization was documented for all procedures. The procedures were performed by several experienced surgeons from the department (certified specialists). The influence of the specialist on the procedure efficacy was not evaluated in this study.

### Follow-up description

Postoperative follow-up was performed in all patients by means of questionnaires and, in most of patients (especially in the modern cohort), uroflowmetry. When the stricture recurrence was considered, ultrasound investigation, urethrography and urethroscopy were performed to aid the diagnosis. Stricture recurrence was defined as the progressive deterioration of the voiding based on objective symptom assessment using International Prostate Symptom Score (IPSS) questionnaire and visualization of the stricture with cystoscopy and cystography with more than 30% of urethral lumen obstruction. Self or assisted dilatation was used in some patients to sustain the urethral lumen. However, we do not possess the detailed information on this parameter, which precluded us from including it in this statistical analysis.

### Ethics

IRB approval was not required by our institution due to the retrospective nature of the study.

### Statistics

Statistical analysis was performed using the STATISTICA 8.0 software (StatSoft, Tulsa, USA). All data samples were tested for normality. Pair-wise comparison of the different parameters among clinical groups was performed with the use of parametric and non-parametric methods. A P level < 0.05 was considered statistically significant. Correlation analysis was performed to identify the associations of clinical/perioperative variables with the outcome. Logistic regression, multiple regression and discriminant analysis were used to create a model for the re-stricture rate prediction based on the clinical and perioperative variables.

### Clinical decision algorithm

One of the aims of our study was to develop a clinical decision algorithm based on the analysis of recurrence or success of urethrotomy in different categories of patients with different disease characteristics, which would incorporate the clinical information and allow for selection of proper treatment in individual patients.

## Results

Database of 470 patients from the Cohort II (modern cohort of our study) with full raw data Median follow-up for patients in Cohort I was 96 months and in Cohort II – 64 months. Click here for additional data file. Copyright: © 2017 Tolkach Y et al. 2017 Data associated with the article are available under the terms of the Creative Commons Zero "No rights reserved" data waiver (CC0 1.0 Public domain dedication).

### Patient demographics and stricture characteristics

The patient demographics and stricture characteristics are listed in Table 1 and Table 2.

**Table 1.** Patient demographics in two cohorts (overall n=961).

	Number of patients	Mean age at diagnosis	Age distribution, years (and percents)									
			0–10	11–20	21–30	31–40	41–50	51–60	61–70	71–80	81–90	91–100
Cohort I	491	59.75±13.7	1	14	38	32	30	87	133	130	25	1
			0.2%	2.9%	7.7%	6.5%	6.1%	17.7%	27.1%	26.5%	5.1%	0.2%
Cohort II	470	61.0±15.6	0	2	29	36	31	67	164	113	24	4
			0%	0.4%	6.2%	7.7%	6.6%	14.3%	34.9%	24.0%	5.1%	0.9%
Total	961	n/a	1	16	67	68	61	154	297	243	49	5
			0.1%	1.7%	7.0%	7.1%	6.3%	16.0%	30.9%	25.3%	5.1%	0.5%

Comments: Age distribution difference between Cohorts I and II was not statistically significant (p>0.05, chi–square test)

**Table 2.** Stricture characteristics in the two cohorts of patients (historical and modern).

	Number pts. available for analysis	Etiology														
		TUR prostate		Idiopathic		Trauma		Hereditary		Urethroplasty		RPE		Infection		Catheterization
Cohort I	366	157		127		19		0		0		0		12		51
		42.9%		34.7%		5.2%		0.0%		0.0%		0.0%		3.3%		13.9%
Cohort II	470	134		176		16		1		13		79		10		41
		28.5%		37.4%		3.4%		0.2%		2.8%		16.8%		2.1%		8.7%
		Location														
		Penile		Prebulbar		Bulbar		Anastomosis (RPE)			Bladder neck		Combination*		Unclear	
Cohort I	491	132		65		148		0			0		117		29	
		26.9%		13.2%		30.1%		0.0%			0.0%		23.8%		5.9%	
Cohort II	470	124		54		230		20			8		10		24	
		26.4%		11.5%		48.9%		4.3%			1.7%		2.1%		5.1%	
		Number of strictures						Number of pts.			Length of stricture					
		1		2		3					≤1 cm		1–2 cm		no data	
Cohort I	421	262		159 ^§^				491			150		176		165	
		62%		38%							30.5%		35.8%		33.6%	
Cohort II	470	406		47		17		470			110		151		209	
		86.4%		10%		3.6%					23.4%		32.1%		44.5%	
		Duration of catheterization. days														
		**1**	**2**	**3**	**4**	**5**	**6**	**7**	**8**	**9**	**10**	**11**	**12**	**13**	**14**	**>14**
Cohort I	316	213	31	27	11	5	0	6	0	2	4	0	0	0	1	16
p<0.01		67,4%	9.8%	8.5%	3.5%	1.6%	0.0%	1.9%	0.0%	0.6%	1.3%	0.0%	0.0%	0.0%	0.3%	5.1%
Cohort II	441	3	46	109	86	79	38	22	14	4	5	8	3	3	3	18
		0.7%	10.4%	24.7%	19.5%	17.9%	8.6%	5.0%	3.2%	0.9%	1.1%	1.8%	0.7%	0.7%	0.7%	4.1%

Comments: TUR – transurethral resection, RPE – radical prostatectomy, * – combination means that more than 2 anatomical segments are affected. § – in our historical cohort the information on the number of strictures by multiple strictures was available only in selected patients. Therefore we show these cases jointly.

The median time for recurrence after initial urethrotomy was 1 year (range 0–9 years), with 33.0% of cases recurring during the first year, 26.4% during the second year and 18.9% during third year. After the second urethrotomy, 48.2% of recurring events were registered during the first year, with 18.5% and 14.3% recurring during the second and third years postprocedure, respectively.

The age distribution of the patients in two cohorts was comparable (p=0.16). More than 70% of patients in both cohorts were 41–80 years old and most were in the 61–70-year-old group. This was reflected in the stricture etiology data ( Table 2) with a prevalence of the strictures, related to prostatic operations (transurethral resection of prostate and radical prostatectomy), which are usually the case in the aforementioned age group.

One of the main differences between both cohorts, which might influence the outcomes, is the catheterization time, and most patients in the historical cohort were on a catheter for fewer than 3 days (67.4% only 1 day) with 3 days and more days in approx. 90% of patients in contemporary group. This parameter was further analyzed as a prognostic factor for the success/failure of the urethrotomy in patients with urethral strictures.

In our analysis, especially related to the development of a clinical decision algorithm, we used the second cohort, which is better investigated and fully supported by clinical and radiological data, while cohort I was used as reference and control for some critical issues that arose during the analysis in the modern cohort.

### Stricture recurrence rates

**Figure 1.**
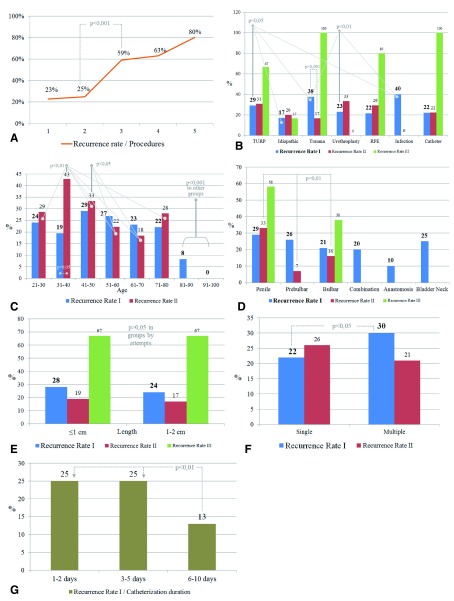
Recurrence rate after optical internal urethrotomy in patients with urethral stricture disease (Cohort II). **A** - Recurrence rate with respect to the number of urethrotomies in each patient. **B** - Recurrence rate with respect to the etiology after the 1 ^st^, 2 ^nd^ and 3 ^rd^ urethrotomies. **C** - Recurrence rate with respect to the patient age after the 1 ^st^ and 2 ^nd^ urethrotomies. **D** - Recurrence rate with respect to the stricture location and number of consecutive procedures. **E** - Recurrence rate with respect to the stricture length (≤1 cm and 1–2 cm) and number of urethrotomies. **F** - Recurrence rate with respect to the number of consecutive strictures in each patient after the 1 ^st^ and 2 ^nd^ attempts. **G** - Recurrence rate with respect to the catheterization duration after the 1 ^st^ urethrotomy.

The overall recurrence rates for cohort II, according to the number of consecutively performed urethrotomies, can be observed in Figure 1A. For this cohort, it was demonstrated that the first and second operation had similar recurrence rates. The recurrence rate significantly increased after the third procedure (p<0.001). The overall recurrence rate after the first operation in cohort I was 32.4% (159 out of 491 patients), which was slightly higher than for Cohort II (23%).

When the stricture etiology was considered ( Figure 1B), differences in the recurrence rates were identified in Cohort II, and there was the highest recurrence rate after the first operation in patients with traumatic lesions and strictures of infectious origin. Interestingly, only 1 out of 6 patients with failure of the first urethrotomy (initial n=16) recurred after the second treatment in the trauma group. No other etiological group was associated with an improved success rate following the second operation. In contrast with all other etiological groups with a generally unfavorable course, patients with idiopathic disease had a stable recurrence rate from the first to third procedure (where the number of cases was sufficient to show a tendency). In Cohort I ( Figure 3-A), a similar success level was detected for the first urethrotomy with the exception of a low recurrence rate for the infection-related strictures. For the second attempt, a substantial increase of the recurrence rate was observed in all etiology groups, except for strictures related to the catheterization.

**Figure 2.**
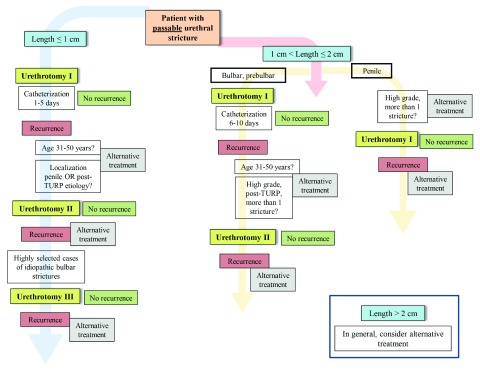
Clinical decision algorithm for patient selection for internal optical urethrotomy according to the clinical parameters and stricture characteristics and ideal time of catheterization (with exclusion of the traumatic strictures and post-prostatectomy anastomotic strictures).

**Figure 3.**
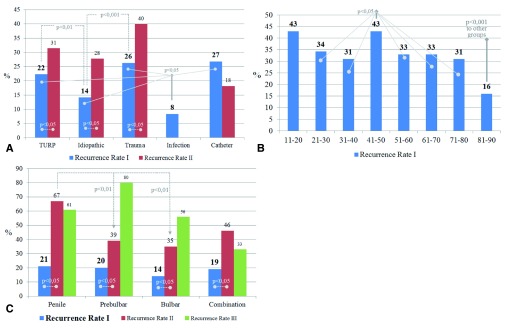
Recurrence rate after optical internal urethrotomy in patients with urethral stricture disease (Cohort I). **A** - Recurrence rate with respect to the etiology after the 1 ^st^ and 2 ^nd^ urethrotomies. **B** - Recurrence rate with respect to the patient age after the 1 ^st^ urethrotomy. **C** - Recurrence rate with respect to the stricture location and number of consecutive procedures (1 ^st^, 2 ^nd^ and 3 ^rd^).

The recurrence rates in Cohort II after the first and second urethrotomy were analyzed with respect to the age of patients ( Figure 1C). Due to inadequate numbers of patients, the age-dependent outcomes of the further treatment attempts were not analyzed. Importantly the lowest recurrence rate after the first procedure was in the 81–90 (only 2 out of 24 patients, 8%) and >90-year-old groups (0 out of 4 patients, 0%) with an overall trend of 19–29% in younger patients without statistical significance between both groups. However, significant differences that negatively affect the success rate after second urethrotomy were observed for the 31–40 and 41–50-year-old groups, demonstrating that the second treatment attempt was by far less successful in those patients. In the Cohort I controls ( Figure 3-B), the same trend was observed favoring the 81–90-year-old group, but a significant difference negatively affecting the outcome of the 41–50-year-old group was evident compared to almost all other age groups (all p<0.05).

The location of the stricture in Cohort II appeared in the outcomes of urethrotomies ( Figure 1D), demonstrating an unfavorable course of penile strictures after the second treatment compared to the bulbar and prebulbar strictures. The third operation in penile strictures failed in more than half of the patients. In Cohort I, a slight tendency to increasing recurrence rates after the second treatment of bulbar and prebulbar and a significant increase in penile strictures was observed. Generally, the third treatment attempt was unfavorable for all patients and combination strictures had an intermediate position.

Interestingly, in Cohort II, there were no significant differences in the recurrence rates for patients with a stricture length of 1 cm or less compared to the strictures that were 1–2 cm in length ( Figure 1E). Multiple strictures tended to be more recurrent than single ones after the first and lacked a difference after the second procedure ( Figure 1F). In Cohort I, information on the length and multifocality of strictures showed no influence on the outcome, which could be statistically demonstrated (p>0.05).

One of the important findings in Cohort II is that a prolonged catheterization (6–10 days) tended to be more favorable in terms of recurrence than ultrashort (1–2 days) and short (3–5 days) regimens (p<0.01) ( Figure 1G). On the contrary, of all patients in Cohort I, 67.4% were postoperatively catheterized for only 1 day, and the minority were catheterized for more than 5 days.

The stricture grade (calculated as the percent of the urethral lumen obstruction), available for analysis (n=255 in Cohort I and n=176 in Cohort II), did not influence the outcome after the first urethrotomy (p>0.05).

**Figure 4.**
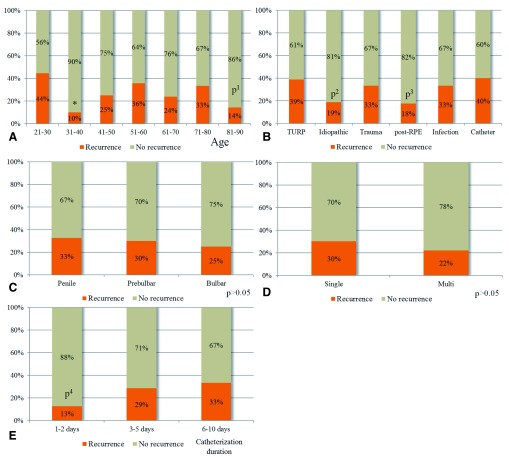
Recurrence rate after the 1 ^st^ optical internal urethrotomy in patients with urethral stricture with a length ≤1 cm. **A** - Recurrence rate with respect to the patient age (*n=5; p ^1^ <0.01). **B** - Recurrence rate with respect to the stricture etiology (p ^2^, p ^3^ < 0.01 compared to the TURP, trauma, infection and catheterization groups). **C** - Recurrence rate with respect to the stricture localization. **D** - Recurrence rate with respect to the number of consecutive strictures in each of the patients. **E** - Recurrence rate with respect to the catheterization duration (p ^4^ <0.05 compared to the other two groups).

**Figure 5.**
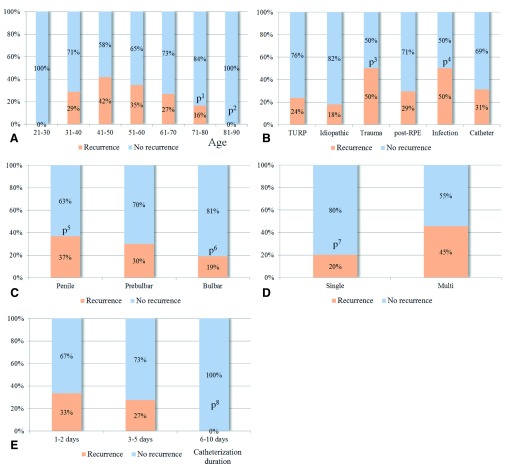
Recurrence rate after the 1 ^st^ optical internal urethrotomy in patients with a urethral stricture with a length of 1–2 cm. **A** - Recurrence rate with respect to the patient age (p ^1^, p ^2^ <0.05 compared to all other groups). **B** - Recurrence rate with respect to the stricture etiology (p ^3^, p ^4^ < 0.01 in comparison to other groups). **C** - Recurrence rate with respect to the stricture localization (p ^5^ <0.01 to bulbar, p ^6^ < 0.05 to prebulbar). **D** - Recurrence rate with respect to the number of consecutive strictures in each of the patients (p ^7^ <0.001). **E** - Recurrence rate with respect to the catheterization duration (p ^8^ <0.01 compared to the other two groups).

Assuming that the stricture length influences the operative outcomes of urethrotomy, we further analyzed the available data from Cohort II ( Figure 4 and Figure 5).

### Recurrence rate analysis in patients with a stricture length of 1 cm or less ( Figure 4)

During the pairwise comparison in patients with or without recurrence after first urethrotomy, there were only slight differences identified. A lower recurrence rate was observed in patients older than 80 years (14% vs. 24–44% in the other age groups, p<0.01; in the 31–40-year-old group, the recurrence rate was 10%, n=5), in patients with idiopathic strictures (19%) and post-radical prostatectomy strictures (18%), but higher recurrence rates were observed in patients with post-TURP strictures (39%), post-traumatic strictures (33%), post-infectious strictures (33%) and strictures related to the catheterization (40%), which had p-levels <0.01. The number of strictures and their stricture localization did not significantly influence the outcome. Patients with ultrashort (1–2 days) catheterization (n=8) had a better success rate (p<0.05). These tendencies were a global trend when all strictures, regardless of length, were considered for analysis.

### Recurrence rate analysis in patients with a stricture length of 1–2 cm ( Figure 5)

When patients with a urethral stricture length greater than 1 cm were considered as a separate group, new factors arose that were important for patient selection in this indication.

The 71–80 and 81–90-year-old groups showed a favorable trend in terms of recurrence after the first procedure, 16% and 0%, respectively, compared to 29–42% in the other groups (p<0.05), except for the 21–30-year-old group in which 9 patients presented with a recurrence rate of 0%. Etiologically, there were no observed advantages of the idiopathic and post-RPE strictures (as was the case in strictures < 1 cm), indicating that the length of a stricture represents a more important factor in these groups. Penile strictures presented with a higher recurrence rate of 37% compared to bulbar strictures (19%; p<0.01). Moreover, the number of strictures seemed to play an important role with a > 2-fold increase in the failure rate in patients with more than 1 versus a singular stricture (45% vs. 20%, p<0.001), and this trend was not present in the patients with short strictures (1 cm or less). The other important finding is the association between the length of catheterization and the success rate of the first procedure. Patients who stayed on a catheter for 6 days or more had a recurrence rate of 0% (n=23) compared to 27% (27 out of 99 patients) in patients who were on a catheter for 5 days and less (p<0.001), indicating that prolonged catheterization influences the outcome of strictures that are 1–2 cm long.

The main clinical questions arising in everyday practice are: Who are the patients who should only be treated once? Who are the patients for whom two attempts could be considered? And who are the patients for whom urethrotomy should never be performed? Further analysis focused on these questions (data from Cohort II) to develop a decision algorithm in patients with stricture disease.

### Who are the patients who should only be treated once and who would benefit from two treatment attempts?

To answer this clinical question, patients with and without recurrence after second stricture treatment were selected and compared to identify factors that were indicative of treatment failure. The most important finding was, that the patients in whom a second operation was successful, had a predominance of bulbar and prebulbar strictures, implying that penile stricture cases are unfavorable for second urethrotomy (recurrence rates of 33%, 7% and 16% for patients with penile, prebulbar and bulbar strictures, p<0.01). Moreover, post-TURP etiology tended to be a greater predictor of failure than other etiological groups (recurrence rates of 31%, 20% and 17% in patients with post-TURP, idiopathic and post-traumatic strictures; p<0.05 for the two latter groups to post-TURP strictures).

Therefore, men with penile strictures and post-TURP etiology are patients in whom any other attempts, except the first, are generally not reasonable.

### Who are the patients, who should never be treated by urethrotomy?

We selected patients from our cohort (n=16) in whom 3 consecutive attempts of urethrotomy were performed with consecutive recurrent strictures, recurred, which represents a group that should initially be treated with other treatment modalities.

Our intention was to identify clinical factors that might be indicative for a successful initial internal urethrotomy. However, besides a trend of a higher number of penile strictures (43.7% of patients in this group) and post-TURP etiology (50% of patients), other parameters were distributed equally compared to the entire study population, providing no answer to this clinical question.

### Prediction models

We have attempted to develop a prediction model based on the database of the Cohort II patients, integrating multiple clinical parameters, such as the age, stricture etiology, length, grade, localization, number of strictures and length of catheterization for predicting the risk of recurrence. Nevertheless, statistical analysis by logistic regression, multiple regression and discriminant analysis did not reveal clear discriminating factors.

## Discussion

Cold-knife direct vision urethrotomy is a technically simple and easy procedure to perform in patients with urethral strictures. As a result, it is the default treatment approach for urethral strictures compared to long-lasting, complex open urethral reconstructions, requiring experience, precise surgical technique, specific instruments and, often, additional materials ^1, 4, 5^. But, the long-term results of urethrotomy are questionable with convincing evidence of high recurrence rates ^2^. Nevertheless, general recommendations about who should undergo urethrotomy and who should not are still lacking ^3^.

We publish results of an analysis in our two consecutive cohorts of patients, who were repeatedly treated with urethrotomy. The high number of patients (n=961) and multiple treatment sessions provide sufficient data for clinical decision-making in patients with urethral strictures.

In the present cohort, some patients had strictures related to the trauma (n=19 and n=16 in Cohorts I and II, respectively) and post-prostatectomy strictures of the vesico-urethral anastomosis (n=20, Cohort II). Both, in our opinion, have to separately be considered due to different endoscopic and other treatment modalities. In case of post-prostatectomy anastomotic strictures, internal urethrotomy or other endoscopic procedures (transurethral resection or laser incision) is the only available treatment modality. These well-established procedures could be combined with experimental techniques, such as glucocorticoid injection in the resection area, with very good overall results ^8– 11^. The data derived from cohort II demonstrated that performing internal urethrotomy in only patients who have an anastomosis stricture achieves a relatively good success rate of 90% after the first urethrotomy ( Figure 1D).

Trauma-related strictures represent a separate clinical problem. Open urethroplasty is considered to be the best treatment at a specialized center of excellence due to the high recurrence rates in case of endoscopic treatment. Moreover, all attempts to perform urethrotomy and other urethral manipulations substantially decrease the success rate of consecutive open urethroplasty ^12– 15^. Only a few patients with short and passable strictures without coarse scarring could be considered for direct vision internal urethrotomy. In our small group of patients with traumatic strictures, the failure rate of the first procedure was relatively high in cohort II (n=16, 38%, p<0.05) and comparable in cohort I (n=19, 26%, p>0.05).

According to our analysis of all other strictures in the anterior urethra, a set of clinical factors influences the outcomes of internal urethrotomy, namely the patient age, stricture etiology, stricture length, number of consecutive strictures in one patient, stricture localization and catheterization duration. These considerations, deriving from the analysis in Cohort II, namely the probability of success and failure of urethrotomy in certain clinical settings (dependent on the characteristics of the stricture disease), allowed us to formulate the clinical decision algorithm for patients with urethral strictures.

### Algorithm of patient selection for urethrotomy and the ideal time of catheterization (traumatic strictures and post-prostatectomy anastomotic strictures excluded) ( Figure 2)

Patients who are 70 years of age and older should be considered as ideal candidates for urethrotomy. The length of the stricture should only be considered in relation to other factors. In patients with short strictures (<1 cm), the etiology, number of strictures and stricture localization did not influence the success rate. The ideal duration of catheterization in this group is 1–5 days (ultra-short catheterization of 1–2 days can be considered). For strictures that are 1–2 cm long, the number of strictures and etiology as well as the duration of catheterization (optimal 6–10 days) significantly influenced the clinical outcomes. Penile strictures (>1 cm) could be treated endoscopically in the presence of a tender stricture. Other treatments should be considered if the number of strictures in those patients exceeds 1. Bulbar strictures with a length of 1–2 cm could be treated endoscopically at least once. Having more than 1 stricture is a predictor of failure. The stricture grade and other parameters should be cautiously considered. A second treatment attempt is generally not recommended in the 31–50-year-old age group. Penile strictures, as well as post-TURP strictures, should only be treated once. All other localizations or etiologies, except multiple long strictures, could be attempted twice. A third attempt should not be performed except for highly selected cases of idiopathic bulbar strictures. Strictures longer than 2 cm should only be considered for an open reconstruction.

Moreover, other factors influenced the outcomes. In the present study, we aimed to create a prognostic model based on the aforementioned clinical parameters. However, it was impossible to identify factors in spite of the clinically significant stricture-related factors. This implies that the factors were randomly distributed throughout the cohort and neither a single nor multiple factors were able to predict the outcome. Therefore, other factors (e.g., severity of spongiofibrosis and individual reactivity) that were not in the scope of this study might be useful for predicting treatment outcomes. Spongiofibrosis, according to several promising exploratory studies and believed to significantly limit success of internal urethrotomy in patients with stricture disease, could be detected pre-operatively by means of magnetic resonance imaging or ultrasound investigation and can therefore be considered with other clinical variables, given that specificity and sensitivity of the diagnostic modality could reach acceptable levels ^16– 18^.

Our algorithm demonstrates that there are some discrepancies with other large, published series. Our analysis shows that in many bulbar and prebulbar strictures, a second urethrotomy, even in case of long strictures up to 2 cm, could be safely attempted with promising success rates. Other authors reported that repeated urethrotomy did not improve the success rate, concluding that only a single procedure should be considered in all patients ^6, 19^. Due to the high failure rates, the treatment of strictures with a length of more than 1 cm by urethrotomy should be avoided in accordance with several studies ^7, 20, 21^. The duration of the postoperative bladder drainage is also controversially discussed ^7, 21^. Nevertheless, in the majority of these studies, the overall success rate was approximately 60%, implying that a more flexible algorithm could extend the indications for direct vision internal urethrotomy, even for disease with recurrent structures. Given that our patients, in case of recurrence, received repeated treatment sessions, we were able to perform a thorough analysis of cases of which repeated urethrotomies were successful, leading to the development of the aforementioned algorithm, providing a therapeutic reserve before these patients were subjected to open urethral reconstruction. Certainly this algorithm needs further investigation in a prospective trial to confirm its applicability and reliability.

Another important issue to consider is that more than 50% of all strictures originate with iatrogenic manipulations (transurethral resection, prostatectomy and catheterization), which should be a serious alert for urologists. This finding substantiates that no safe and easy manipulations on the urethra are available and that the urethra is very sensitive to traumatization, warranting a careful approach.

Our study is not devoid of limitations related to the retrospective nature of data acquisition, possible biases, and the absence or inaccuracy of data in some patients. Particularly, some parameters, which were not available in our patients, namely the use of self or assisted dilatation of the urethra (which could have led to higher success rate of urethrotomy in our study compared to the more strict criteria in other studies), and the surgical experience with urethrotomy of operating surgeons, could certainly make the conclusions more durable. Nevertheless, the retrospective study design and big patient cohort provided extensive valid information for performing a thorough statistical analysis that could be used to generate important issues that could be implemented in our clinical decision algorithm.

## Conclusions

Based on two cohorts of patients, we have performed analysis of the clinical factors related to the efficacy of the primary and repeated urethrotomies in male patients with urethral stricture disease. Based on these findings, a flexible clinical decision algorithm was developed for this group of patients, providing a rationale for the optimal selection of patients for endoscopic treatment.

## Data availability

The data referenced by this article are under copyright with the following copyright statement: Copyright: © 2017 Tolkach Y et al.

Data associated with the article are available under the terms of the Creative Commons Zero "No rights reserved" data waiver (CC0 1.0 Public domain dedication). 

Dataset 1. Database of 470 patients from the Cohort II (modern cohort of our study) with full raw data, 10.5256/f1000research.9427.d135465 ^22^
