# Supplementary material for: Development of a clinical algorithm for treating urethral strictures based on a large retrospective single-center cohort
Source: F1000Res. 2017 Apr 24;5:2378. Originally published 2016 Sep 26. [Version 2] doi: 10.12688/f1000research.9427.2 (PMC5414819; doi:10.12688/f1000research.9427.2)
Supplement: Peer review history

# Peer review: Referee response for version 2

**DOI:** 10.5256/f1000research.12402.r22159

**Published:** 2017-08-01

**Authors:** Bastian Amend

**Affiliations:** 1 Department of Urology, Eberhard Karls University Tuebingen, Tübingen, Germany

The authors submitted their revised manuscript. All my questions and concerns have been answered comprehensively. Congratulations to the scientific work.

I have read this submission. I believe that I have an appropriate level of expertise to confirm that it is of an acceptable scientific standard.

# Peer review: Referee response for version 2

**DOI:** 10.5256/f1000research.12402.r22574

**Published:** 2017-05-08

**Authors:** Margit Fisch, Clemens Rosenbaum, Luis Kluth

**Affiliations:** 1 Department of Urology, University Medical Center Hamburg-Eppendorf, Hamburg, Germany; 2 Department of Urology, University Medical-Center Hamburg-Eppendorf, Hamburg, Germany; 3 Department of Urology, University Hospital Hamburg-Eppendorf, Hamburg, Germany

We have re-reviewed the article and have no further comments. Our concerns have been adequately addressed.

We have read this submission. We believe that we have an appropriate level of expertise to confirm that it is of an acceptable scientific standard.

# Peer review: Referee response for version 2

**DOI:** 10.5256/f1000research.12402.r22158

**Published:** 2017-05-02

**Authors:** Peter F.W.M Rosier

**Affiliations:** 1 Department of Urology, University Medical Center Utrecht, Utrecht, Netherlands

I have no further comments, my concerns have been adequately addressed.

I have read this submission. I believe that I have an appropriate level of expertise to confirm that it is of an acceptable scientific standard.

# Peer review: Referee response for version 1

**DOI:** 10.5256/f1000research.10155.r18345

**Published:** 2016-12-08

**Authors:** Margit Fisch, Clemens Rosenbaum, Luis Kluth

**Affiliations:** 1 Department of Urology, University Medical Center Hamburg-Eppendorf, Hamburg, Germany; 2 Department of Urology, University Medical-Center Hamburg-Eppendorf, Hamburg, Germany; 3 Department of Urology, University Hospital Hamburg-Eppendorf, Hamburg, Germany

The article “Development of a clinical algorithm for treating urethral strictures based on a large retrospective single-center cohort” provides durable data based on a large number of patients treated by direct vision internal urethrotomy (DVIU) for urethral stricture diseases. Aim of this study was to develop an algorithm for clinical decision-making.

**Introduction:**

 As discussed, success rates given by the literature vary broadly. The authors stated it ranges between 32% and 73.1%. This leaves out data given by Santucci et al. with significantly lower success rates between 0 and 9% only, that said, this was a smaller patient cohort ^1^. These data should be included as they mirror the variety in results given by the literature.

 The authors stated: “The guidelines issued by the professional organizations do not generally recommend urethrotomy in patients with strictures longer than 1 cm or repeated urethrotomy sessions.” These guidelines should be quoted ^2^.

**Results/discussion:**

 The time to recurrence of urethral stricture would be interesting to know.

 Vesicourethral anastomosis stenosis (VUS), bladder neck stenosis (BNS) and traumatic posterior urethral stenosis are different to anterior urethral strictures. It should be avoided to mix theses different reasons for bladder outlet obstruction. As partially discussed the 28 (VUS and BNS) and 16 (trauma) patients in the modern cohort should be excluded. If they have been, as it remains unclear after restudying the material and methods as well as the discussion part, it needs to be clarified more precisely.  

 In the material and methods section it is stated: “Only patients without any prior treatment of urethral strictures were included in the study”. However, in the results we have listed 13 patients with prior urethroplasty in the modern cohort (Table 2). These patients should be excluded. Stricture recurrence after urethroplasty is a different situation and should be considered elsewhere ^3^.  

 Further on, data as shown in Figure 1 should be supported by total number of patients analyzed: Data like declining recurrence rates in repeated DVIUs in patients with prebulbar strictures hint at small sample sizes. This issue is addressed in results: “No other etiological group was associated with an improved success rate following the second operation.” But no conclusion has been drawn from this statement. 

 The authors stated: “One of the important findings in Cohort II is that a prolonged catheterization (6–10 days) tended to be more favorable in terms of recurrence than ultrashort (1–2 days) and short (3–5 days) regimens (p<0.01)”. This stands in contrast to published data by Albers et al. with a comparable patient cohort ^4^. This issue needs to be discussed.

 The significant differences negatively affecting the success rate after second DVIU for the 31–50-year-old groups, demonstrating that the second treatment attempt was by far less successful in those patients, needs to be discussed as well. As stated: “The number of strictures and their stricture localization did not significantly influence the outcome”. This again is in contrast to most data given. It needs to be discussed as well.

 In Figure 2, some arrows to illustrating the next suggested treatment (for example: after 1.DVIU in a short stricture in case of recurrence, an arrow to other treatments in case of younger age, penile or post-TURP-strictures) would be helpful to faster understand the figure.

 Overall, the effort made to find a clinical algorithm how and when to apply DVIUs is excellent. In this paper data given by this large cohort seems reliable. Unfortunately, statistical analysis did not reveal clear discriminating factors. This extenuates the power of the proposed algorithm and should be discussed more clearly. It seems obvious that other factors, as discussed by the authors, seem to influence outcome more relevant. As long as these factors are not identified clearly, an algorithm as proposed by the authors seems to be the most applicable tool.

 Based on these findings, this reviewer considers this manuscript as a minor revision.

We have read this submission. We believe that we have an appropriate level of expertise to confirm that it is of an acceptable scientific standard, however we have significant reservations, as outlined above.

## References

[1] SantucciREisenbergL: Urethrotomy has a much lower success rate than previously reported. *J Urol*.2010;183(5) : 10.1016/j.juro.2010.01.020 1859-62 10.1016/j.juro.2010.01.020 20303110

[2] BuckleyJCHeynsCGillingPCarneyJ: SIU/ICUD Consultation on Urethral Strictures: Dilation, internal urethrotomy, and stenting of male anterior urethral strictures. *Urology*.2014;83(3 Suppl) : 10.1016/j.urology.2013.08.075 S18-22 10.1016/j.urology.2013.08.075 24286602

[3] RosenbaumCMSchmidMLudwigTAKluthLAReissPDahlemREngelOChunFKRiechardtSFischMAhyaiSA: Internal urethrotomy in patients with recurrent urethral stricture after buccal mucosa graft urethroplasty. *World J Urol*.2015;33(9) : 10.1007/s00345-014-1450-y 1337-44 10.1007/s00345-014-1450-y 25428791

[4] AlbersPFichtnerJBrühlPMüllerSC: Long-term results of internal urethrotomy. *J Urol*.1996;156(5) :1611-4 8863550

# Peer review: Referee response for version 1

**DOI:** 10.5256/f1000research.10155.r17960

**Published:** 2016-11-29

**Authors:** Bastian Amend

**Affiliations:** 1 Department of Urology, Eberhard Karls University Tuebingen, Tübingen, Germany

The presented study is based retrospectively on a considerable patient population. This makes conclusive statements possible.

 Some questions need revision/to be answered: The time to recurrence of urethral stricture would be necessary to know. In addition, it might be interesting to see whether the specialist status has an influence on the recurrence rate. Have patients with laser urethrotomy been excluded?

 Overall the idea of a flow chart as a result of data analysis is excellent and worthful for clinical daily practice.

I have read this submission. I believe that I have an appropriate level of expertise to confirm that it is of an acceptable scientific standard, however I have significant reservations, as outlined above.

# Response

**Published:** 2017-04-20

**Authors:** Yuri Tolkach

**Affiliations:** Institute of Pathology, University Hospital of Bonn, Germany

Dear Dr. Amend, 

Thank you very much for your comment. 

Indeed, a time to recurrence is a very important parameter. We have added the information to the time to recurrence into the Results section of the Manuscript.

Our study does not include the patients with laser urethrotomy, concentrating on the optical cold-knife urethrotomy. We have stated this explicitly in the Materials and Methods. 

In the Materials and Methods we have also clarified that the specialist status was not separately assessed in our study. We agree that this is always a very interesting and understudied confounder of the procedure success, which although very hard to define and evaluate. 

Once again thank you for you time and critical review,

On behalf of all authors,

Yuri Tolkach and Florian Imkamp

# Peer review: Referee response for version 1

**DOI:** 10.5256/f1000research.10155.r16943

**Published:** 2016-10-24

**Authors:** Peter F.W.M Rosier

**Affiliations:** 1 Department of Urology, University Medical Center Utrecht, Utrecht, Netherlands

The paper provides a detailed overview of single center data. I slightly miss 'time to recurrence' and basically the endeavour remains without the result that was aimed. Few other issues are that all information about self dilatiation misses. And also that it is unclear what the definition of 'traumatic stricture' has been, as separate from 'unknown'. Last but not least the single center results could be much better integrated in the body of evidence that exists for this topic.

I have read this submission. I believe that I have an appropriate level of expertise to confirm that it is of an acceptable scientific standard, however I have significant reservations, as outlined above.

# Response

**Published:** 2017-04-20

**Authors:** Yuri Tolkach

**Affiliations:** Institute of Pathology, University Hospital of Bonn, Germany

Dear Dr. Rosier,

We are very thankful for your input and review. 

We have added the time to recurrence information in the Results section of the Manuscript. 

We are agree that the self dilatation is a very important parameter to analyse, however in our cohort this data was only partially available, making it's inclusion into analysis impossible. We have stated this explicitly in the Materials and Methods and also in the Discussion of the limitations of our article. It's indeed could lead to somehow higher rates of the success for urethrotomy, the effect which we also state as limitation of our study. 

Moreover, we have provided the definition of the "traumatic stricture" used in our study (in Materials and Methods).

Thank you very much for efforts,

With kind regards, 

on behalf of all authors,

Yuri Tolkach and Florian Imkamp
